# Whole-Transcriptome Sequencing of Antler Tissue Reveals That circRNA2829 Regulates Chondrocyte Proliferation and Differentiation via the miR-4286-R+1/FOXO4 Axis

**DOI:** 10.3390/ijms24087204

**Published:** 2023-04-13

**Authors:** Haibo Yao, Renfeng Jiang, Danyang Chen, Yanjun Li, Mengmeng Song, Zitong Sun, Guohui Long, Lei Wu, Wei Hu

**Affiliations:** College of Life Science, Jilin Agriculture University, Changchun 130118, China; hiber9@126.com (H.Y.);

**Keywords:** sika deer, antler, RNA sequencing, ceRNAs, circRNA2829, miR-4286-R+1, FOXO4

## Abstract

The antler is the unique mammalian organ found to be able to regenerate completely and periodically after loss, and the continuous proliferation and differentiation of mesenchymal cells and chondrocytes together complete the regeneration of the antler. Circular non-coding RNAs (circRNAs) are considered to be important non-coding RNAs that regulate body development and growth. However, there are no reports on circRNAs regulating the antler regeneration process. In this study, full-transcriptome high-throughput sequencing was performed on sika deer antler interstitial and cartilage tissues, and the sequencing results were verified and analyzed. The competing endogenous RNA (ceRNA) network related to antler growth and regeneration was further constructed, and the differentially expressed circRNA2829 was screened out from the network to study its effect on chondrocyte proliferation and differentiation. The results indicated that circRNA2829 promoted cell proliferation and increased the level of intracellular ALP. The analysis of RT-qPCR and Western blot demonstrated that the mRNA and protein expression levels of genes involved in differentiation rose. These data revealed that circRNAs play a crucial regulatory role in deer antler regeneration and development. CircRNA2829 might regulate the antler regeneration process through miR-4286-R+1/FOXO4.

## 1. Introduction

The antler is the incompletely ossified horn of the deer family, which has a unique charm as a bony organ with an extremely fast growth rate and capacity for complete regeneration [1]. Antler growth is mainly triggered by a growth center composed of mesenchyme and cartilage and contains a series of biological processes, such as wound repair, cell proliferation, vascular regeneration, hormonal regulation, tissue transformation, cartilage ossification, and epidermal exfoliation [2,3,4]. These complex biological processes are collectively involved in antler regeneration, and many factors influence and regulate antler regeneration. Therefore, the mechanisms of antler regeneration have not been fully elucidated. These features have also led to a high level of interest in the mystery of antler regeneration.

In recent years, there have been increasing reports on the regulatory roles of non-coding RNAs (ncRNAs) in related biological processes. Some miRNAs and lncRNAs that play vital roles in disease genesis and organ development have been identified [5,6]. With the development of high-throughput sequencing technology, significant progress has been made in studies related to circular RNAs (circRNAs) [7,8]. Unlike linear RNAs, circRNAs lack a 5′ end cap structure and a 3′ end polyadenylate tail and form a special covalent closed-loop structure by connecting their head and tail, through covalent bonds [9]. CircRNA is not easily degraded by exonuclease and is more stable than linear RNA. Recent studies have shown that circRNA molecules act as miRNA sponges in cells, thereby relieving the inhibition effect of miRNA on its target genes and increasing the expression level of target genes. This mechanism of action is called the competitive endogenous RNA (ceRNA) mechanism [10,11,12]. Compared to miRNA regulatory networks, ceRNA networks, as a novel mode of gene expression regulation, are more elaborate and complex, involving more RNA molecules. Therefore, this is a meaningful way to study the regulatory role of ncRNAs in vivo [13].

In this study, whole-transcriptome high-throughput sequencing of antler tip tissues (mesenchymal and cartilage) confirmed the presence of a large number of differentially expressed ncRNAs in antlers, which provides a new theoretical basis for exploring the regulation of ncRNAs on antler proliferation and regeneration. To deeply investigate the regulatory mechanism of circRNAs in antler regeneration, we identified a new circRNA2829 differentially expressed in two tissues through a series of screenings, and revealed the function of circRNA2829 in regulating chondrocyte proliferation and differentiation through the miR-4286-R+1/FOXO4 axis by systematic experiments and interaction mechanisms. Overall, these findings provide new insights into the role of circRNAs in revealing the mechanisms of antler regeneration and rapid growth.

## 2. Result

### 2.1. Analysis of the Differentially Expressed circRNAs, miRNAs, and mRNAs in Tip Tissue of Antlers

**Figure 1:**
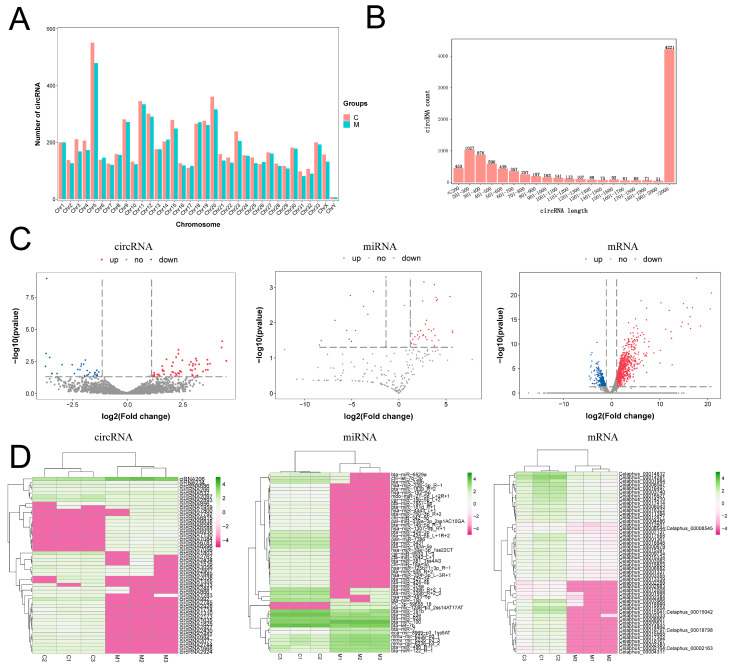
Analysis of the differentially expressed circRNA, miRNA, and mRNA in tip tissue of antlers. (**A**) circRNA distribution on chromosomes. (**B**) The length distribution of circRNA. (**C**) Volcano diagrams of differential circRNA, miRNA, and mRNA. DEGs were selected with an absolute value of the thresholds of fold change ≥1 and *p* < 0.05, the red dots represent upregulated differential genes, the blue dots represent downregulated differential genes, and the black dots represent genes with no significant difference. (**D**) Clustering heatmap of the top 50 differentially expressed circRNA, miRNA, and mRNA; the pink represents downregulated and the green represents upregulated.

The distribution of circRNAs on chromosomes and the length of circRNAs were analyzed. The number of circRNAs was significantly higher in chromosome5 (Chr5) than in other chromosome groups (Figure 1A). The size of the circRNAs ranged from 72 nt to 197600 nt (Figure 1B). The results of the analysis with Limma package and DEseq2 revealed that the RNA molecules, including 175 circRNAs (116 upregulated, 59 downregulated), 197 miRNAs (172 upregulated, 25 downregulated), and 1488 mRNAs (915 upregulated, 573 downregulated), were differentially expressed in both tissues. A volcano map (Figure 1C) and cluster heat map (Figure 1D) were used to visualize the results.

### 2.2. Validation of High-Throughput Sequencing Results

**Figure 2:**
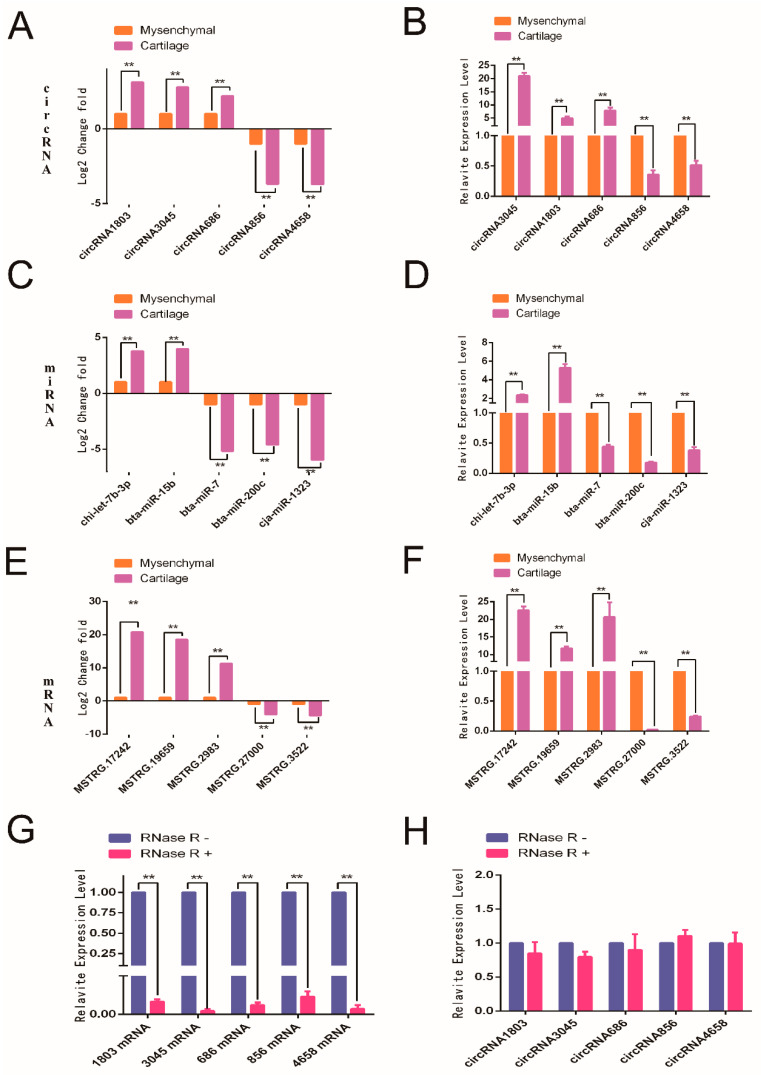
Validation of high-throughput sequencing results. (**A**,**C**,**E**) Analysis of the expression levels of circRNA, miRNAs, and mRNAs in high-throughput sequencing results. (**B**,**D**,**F**) RT-qPCR validation results of circRNA, miRNAs, and mRNAs. (**G**,**H**) RNase R digestion followed by RT-qPCR of circRNA and their host genes. ** *p* < 0.01.

RT-qPCR validation was performed on five randomly selected circRNAs, miRNAs, and mRNAs, and the results of the RT-qPCR were found to be consistent with the results of the high-throughput sequencing (Figure 2A–F). In addition, we also designed RNase R treatment experiments to verify the presence of the circular structure of circRNAs in the high-throughput sequencing results. The RT-qPCR results suggested that randomly selected circRNAs were more stable than mRNAs (Figure 2G,H).

### 2.3. GO and KEGG Analysis of circRNAs and mRNAs

**Figure 3:**
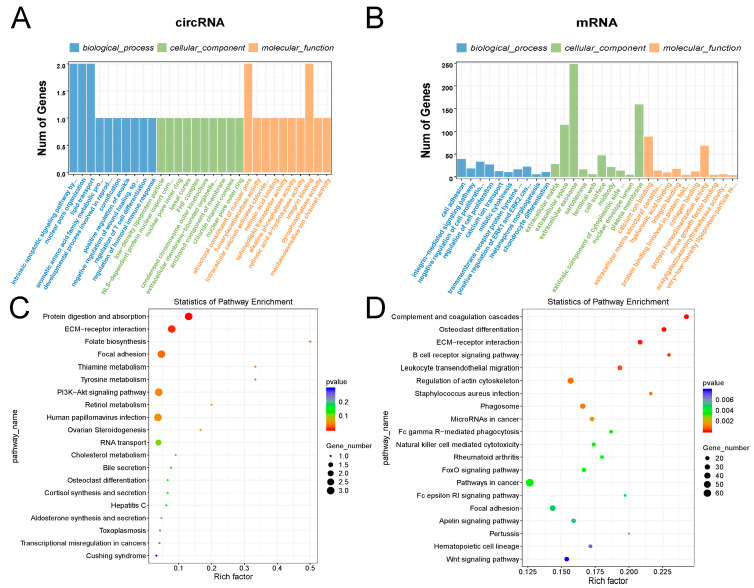
GO and KEGG analysis of circRNA, miRNA, and mRNA. (**A**,**B**) Top 10 GO terms for differentially expressed circRNA and mRNAs in biological processes, cellular components, and molecular functions. (**C**,**D**) KEGG analysis of differentially expressed circRNA and mRNAs. Only the top 10 enriched KEGG pathways are shown in the figure.

The GO and KEGG results indicated that the GO terms that differentially expressed circRNAs were mainly enriched in p53-like mediators of the intrinsic apoptotic signaling pathway, nuclear pore organization, lipid transport, the structural composition of nuclear pores, and integrin binding proteins, and the KEGG pathway mainly included protein treatment and uptake, ECM-receptor interaction, and the PI3K-Akt signaling pathway (Figure 3A,C). The GO terms that differentially expressed mRNAs were mainly enriched in cell proliferation, cell differentiation, and cellular exosomes, and the KEGG pathway mainly included complement and coagulation cascade response, osteoclast differentiation, and ECM-receptor interaction (Figure 3B,D).

### 2.4. Construction and Analysis of circRNA-miRNA-mRNA ceRNA Network

**Figure 4:**
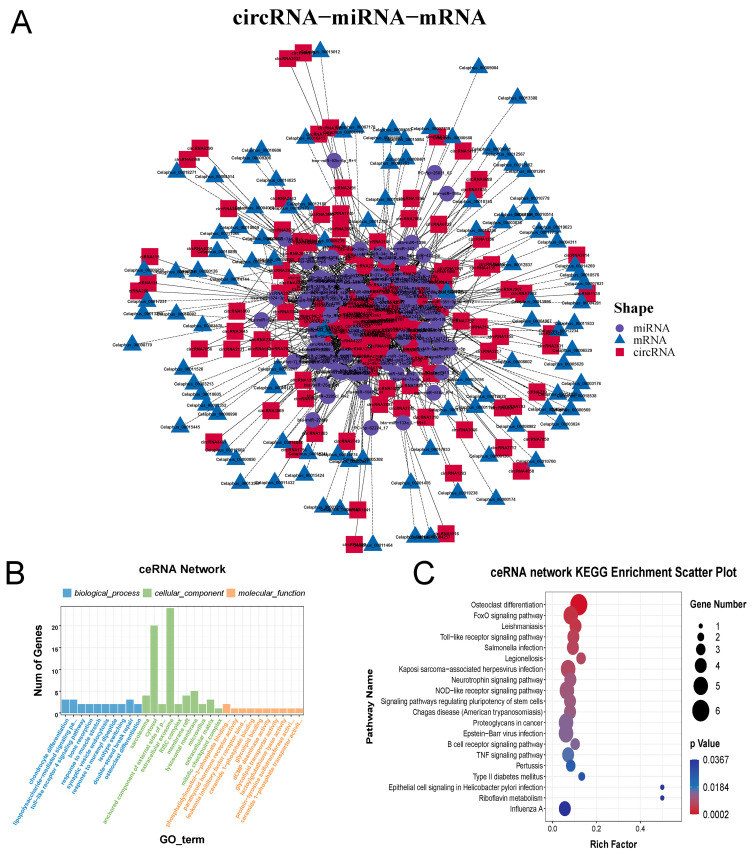
Construction and Analysis of circRNA-miRNA-mRNA ceRNA Network. (**A**) Regulatory networks of circRNA-miRNA-mRNA interactions in mesenchymal and cartilage tissue of antlers. (**B**) The top 10 GO terms of the ceRNA network in biological process, cellular component, and molecular function. (**C**) KEGG analysis of the pathways of the ceRNA network. The figure shows only the top 10 enrichment KEGG pathways.

A circRNA-miRNA-mRNA ceRNA network was constructed using TargetScan (5.0a) and Miranda (3.3a) to predict the target binding relationships of circRNA, miRNA, and mRNA in mesenchymal and cartilage tissues (Figure 4A). GO and KEGG enrichment analysis were performed on the RNAs in the ceRNA network. The results revealed that the mRNAs in the ceRNA network were mainly enriched for GO terms including chondrocyte differentiation, the toll-like receptor 4 signaling pathway, synaptic vesicle endocytosis, double-strand break repair, and osteoclast differentiation (Figure 4B). The enriched KEGG pathway included osteoclast differentiation, the FOXO signaling pathway, the Toll-like receptor signaling pathway and the signaling pathway regulating stem cell pluripotency (Figure 4C). 

### 2.5. CircRNA2829 Was Identified as a Circular RNA Related to Antler Regeneration and Differentiation

**Figure 5:**
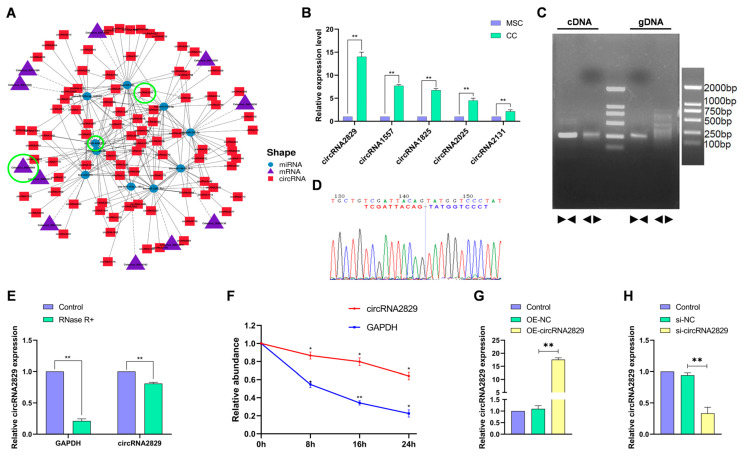
circRNA2829 was identified as a circular RNA related to antler regeneration and differentiation. (**A**) The circRNA-miRNA-mRNA interaction network is related to the development and regeneration of antlers. (**B**) The top five circRNA in the ceRNA network with relative expression levels were detected by RT-qPCR. (**C**) The agarose gel electrophoresis was performed using divergent primers and convergent primers to verify the existence and circular structure of circRNA2829. (**D**) Sanger sequencing was used to validate that the back splice site existed. (**E**,**F**) Relative expression levels of GAPDH and circRNA2829 after RNase R treatment or after Act-D treatment were examined by RT-qPCR. (**G**,**H**) Relative expression levels of circRNA2829 were detected by RT-qPCR after treatment of chondrocytes with OE-circRNA2829 or si-circRNA2829. ** *p* < 0.01, * *p* < 0.05.

To further investigate the differentially expressed ceRNAs associated with antler growth and regeneration in mesenchymal and cartilage tissues, we screened the ceRNAs associated with GO terms such as cartilage development, cartilage agglutination, bone resorption, bone mineralization, and osteoblast development and mapped the ceRNA network (Figure 5A). The top five circRNAs in the network with the highest expression levels were selected for RT-qPCR analysis, and circRNA2829 was found to have the highest expression level (Figure 5B). PCR assays and the agarose gel electrophoresis results revealed that convergent primers amplified a band of the same length in both cDNA and genomic DNA (gDNA). However, the divergent primers only amplified bands in cDNA but not in gDNA (Figure 5C). Sanger sequencing confirmed the back-splice junction (Figure 5D). RNase R treatment experiments and Act-D treatment experiments further verified the stability of circRNA2829 compared to linear GAPDH (Figure 5E,F). Next, over-expression circRNA2829 (OE-circRNA2829) or si-circRNA2829 was transfected into chondrocytes, and the RT-qPCR results confirmed that circRNA2829 was successfully overexpressed or interference compared to the control (Figure 3G,H).

### 2.6. CircRNA2829 Promotes Chondrocyte Proliferation and Differentiation

**Figure 6:**
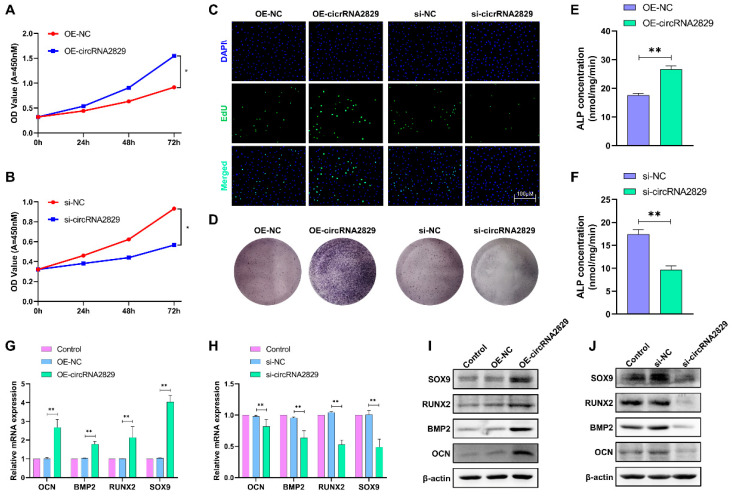
circRNA2829 promotes chondrocyte proliferation and osteogenic differentiation. After transfection of OE-circRNA2829 or si-circRNA2829 in chondrocytes, CCK-8 (**A**,**B**) and EdU (**C**) were used to determine cell proliferation ability; BCIP/NBT staining (**D**) and ALP viability assay (**E**,**F**) to assess the osteogenic differentiation ability of chondrocytes; RT-qPCR (**G**,**H**) and Western blot (**I**,**J**) to detect changes in the transcriptional and translational levels of osteogenic marker-related genes. ** *p* < 0.01, * *p* < 0.05.

To study the effect of circRNA2829 on chondrocyte proliferation and differentiation, we treated chondrocytes with OE-circRNA2829 and si-circRNA2829. The results of cell counting kit-8 (CCK-8) and 5-ethynyl-2′-deoxyuridine (EdU) experiments demonstrated that the overexpression of circRNA2829 significantly promoted chondrocyte proliferation, while the downregulation of circRNA2829 inhibited the proliferation of chondrocytes (Figure 6A–C). ALP staining showed a significant increase in stained cells in the OE-circRNA2829 group compared to the control group, while si-circRNA2829 caused a decrease in stained cells (Figure 6D). In addition, the results of the ALP activity assay also confirmed that OE-circRNA2829 increased ALP levels in chondrocytes, while si-circRNA2829 decreased ALP levels in chondrocytes (Figure 6E,F). Finally, the changes in osteogenic marker genes and proteins (OCN, BMP2, RUNX2, SOX9) in chondrocytes after transfection with circRNA2829 were detected by RT-qPCR and the Western blot assay (Figure 6G–J). The results indicated that the expression levels of osteogenic-related genes and their proteins increased after the circRNA2829 overexpression, while the si-circRNA2829 group produced the opposite experimental results. These results confirm that circRNA2829 plays an essential role in promoting the proliferation and differentiation of chondrocytes, thus promoting the rapid growth and regeneration of antlers.

### 2.7. MiR-4286-R+1 Inhibits Chondrocyte Proliferation and Differentiation

**Figure 7:**
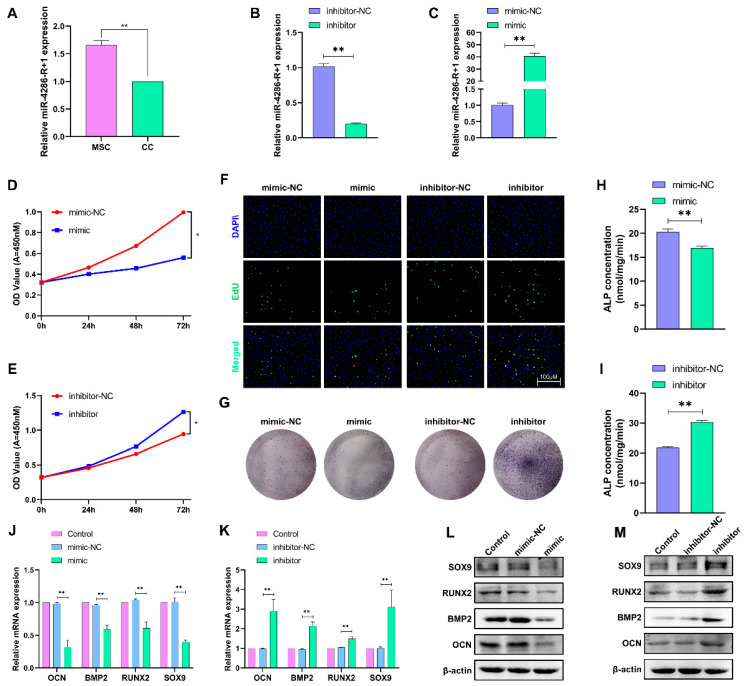
MiR-4286-R+1 inhibits chondrocyte proliferation and osteogenic differentiation. (**A**,**B**) Detection of miR-4286-R+1 expression level after transfection with mimic or inhibitor by RT-qPCR. (**C**) RT-qPCR detection of si-FOXO4 interference efficiency. After transfection of miR-4286-R+1 mimic or inhibitor in chondrocytes, CCK-8 (**D**,**E**) and EdU (**F**) were used to determine cell proliferation ability; BCIP/NBT staining (**G**) and ALP viability assay (**H**,**I**) to assess the osteogenic differentiation ability of chondrocytes; RT-qPCR (**J**,**K**) and Western blot (**L**,**M**) to detect changes in the transcriptional and translational levels of osteogenic marker-related genes. ** *p* < 0.01, * *p* < 0.05.

MiR4286-R+1 is predicted to be a key target miRNA for circRNA2829, and we subsequently explored the expression pattern and function of miR4286-R+1 in chondrocytes. The RT-qPCR analysis displayed that the miR4286-R+1 expression was significantly downregulated in chondrocytes compared to mesenchymal stem cells (Figure 7A). Next, the overexpression or interference of miRNA4286-R+1 was verified by RT-qPCR in chondrocytes transfected with a mimic and inhibitor (Figure 7B,C). In the detection of CCK-8 and EdU, the mimic was found to inhibit chondrocyte proliferation significantly. In contrast, treatment with an inhibitor promoted chondrocyte proliferation (Figure 7D–F). Similarly, the results of staining cells using a BCIP/NBT reagent and intracellular ALP activity indicated that miR4286-R+1 inhibited the differentiation ability of chondrocytes (Figure 7G–I). The Western blot assay and RT-qPCR experiments confirmed that the mimic decreased the expression levels of osteogenic marker genes and proteins, while the inhibitor produced the opposite results (Figure 7J–M).

### 2.8. CircRNA2829 Promotes Chondrocyte Proliferation and Differentiation via the miR4286-R+1/FOXO4 Axis

**Figure 8:**
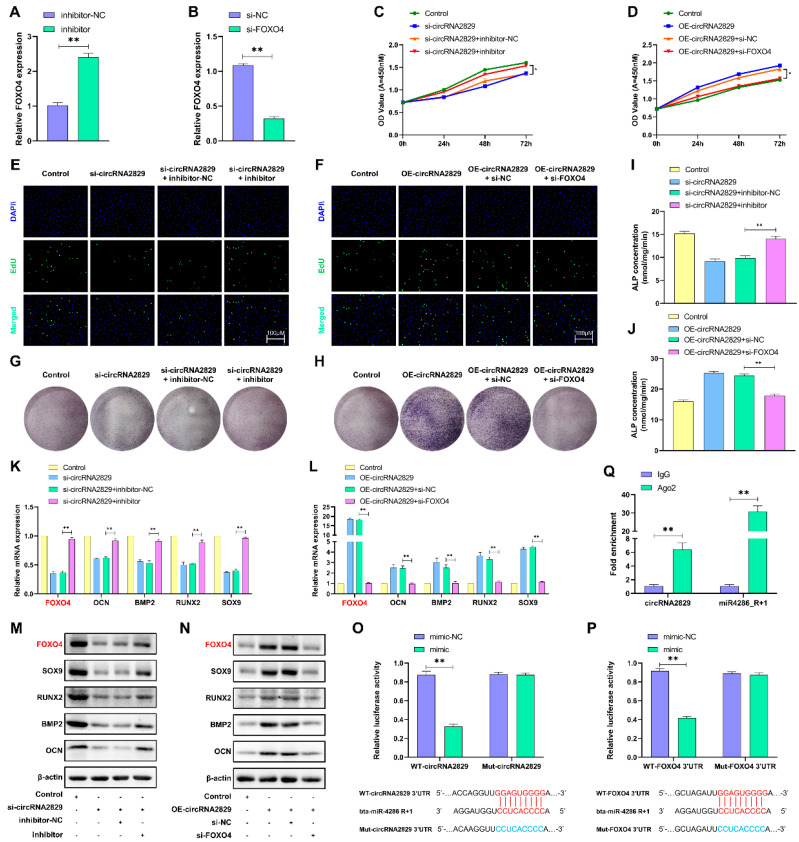
circRNA2829 promotes chondrocyte proliferation and differentiation via the miR4286-R+1/FOXO4 axis. (**A**,**B**) RT-qPCR to detect changes in FOXO4 expression levels in chondrocytes after transfection with inhibitor or si-FOXO4. After cotransfection of si-circRNA and inhibitor or cotransfection of OE-circRNA and si-FOXO4 in chondrocytes, cell proliferation ability was measured by CCK-8 (**C**,**D**) and EdU (**E**,**F**); osteogenic differentiation ability of chondrocytes was assessed by BCIP/NBT staining (**G**,**H**) and ALP viability assay (**I**,**J**); RT-qPCR (**K**,**L**) and Western blotting (**M**,**N**) were used to detect changes in the mRNA and protein of osteogenic marker-related genes. (**O**,**P**) Dual luciferase reporter gene assay to demonstrate the binding site of miR-4286-R+1 to circRNA2829 and FOXO4; (**Q**) RT-qPCR to detect the expression levels of circRNA2829 and miR-4286-R+1 in RIP antibody complexes. ** *p* < 0.01, * *p* < 0.05.

Using the TargetScan online tool and Miranda online database, FOXO4 was identified as a direct target gene of miR4286-R+1. After transfection of the miR4286-R+1 inhibitor and si-FOXO4 in chondrocytes, the RT-qPCR results showed downregulation of FOXO4 expression levels (Figure 8A,B). Based on these results, we next explored whether the function of circRNA2829 interacts with the miR4286-R+1/FOXO4 axis and functions in cartilage. Two sets of rescue experiments were set up to demonstrate the interaction between circRNA2829, miRNA-4286-R+1, and FOXO4. The results of the CCK-8 and EdU assays suggested that the inhibition of cell proliferation caused by si-circRNA2829 is counteracted by the miR-4286-R+1 inhibitor (Figure 8C,E). The results of the ALP staining and ALP content assay indicated that the miR-4286-R+1 inhibitor counteracted the effect of si-circRNA2829 (Figure 8G,I). The RT-qPCR and Western blot experiments confirmed that the miR4286-R+1 inhibitor counteracted the impact of si-circRNA2829 on cellular osteogenic marker genes and FOXO4 (Figure 8K,M). Similarly, the promotional effect of OE-circRNA2829 on cell proliferation and differentiation ability was counteracted by si-FOXO4 (Figure 8D,F,H,J,L,N). The above results suggest that there is an interrelationship between circRNA2829, miR4286-R+1 and FOXO4. Finally, the binding sites of circRNA2829 and FOXO4 to miR-4286-R+1 were verified by a luciferase reporter gene assay. As expected, the miR-4286-R+1 mimic significantly inhibited the WT-circRNA2829 and WT-FOXO4 3’UTR reporter luciferase activity, while the MUT-circRNA2829 and MUT-FOXO4 3’UTR reporter activities were not significant between the miR-4286-R+1 mimic and control differences (Figure 8O,P). Immunoprecipitation of Ago2-associated RNA (Ago2-RIP) was performed to pull down endogenous miRNAs and circRNAs bound to Ago2. The RT-qPCR detected that circRNA2829 and miR-4286-R+1 were significantly enriched. 

## 3. Discussion

The differentiation of mesenchymal tissue at the top of the antler into cartilage tissue is a key link in antler regeneration [14]. During antler growth, mesenchymal stem cells continue to proliferate while gradually differentiating into chondrocytes, and chondrocytes re-ossify, alternatively constituting antler growth. Therefore, antler growth depends on the proliferation and differentiation of antler mesenchymal and chondrogenic tissues. With the advancement of RNAomics research, more and more non-coding RNAs have been gradually discovered for their biological functions in the organism. Our previous work has preliminarily demonstrated that miRNAs and lncRNAs have important regulatory roles in the proliferation and differentiation of antler chondrocytes [15,16,17]. Circular non-coding RNAs (circRNAs) are widely present in a variety of cells and tissues and are characterized by structural stability, sequence conservation, and tissue-specific expression, and are involved in a variety of biological processes [18,19,20]. Given this, we performed a whole-transcriptome sequencing of antler tip tissues, to combine multiple RNA information for integrated analysis and explore potential regulatory network mechanisms during antler regeneration and development.

First, we analyzed and validated the differentially expressed circRNAs and mRNAs based on the sequencing results, and found that the biological processes of differentially expressed circRNAs mainly included the p53 intrinsic apoptotic signaling pathway and PI3K-Akt signaling pathway, while mRNAs were mainly enriched in osteoclast differentiation, ECM-receptor interaction, the B-cell receptor signaling pathway, cell transendothelial migration, and regulation of the actin cytoskeleton. These results suggest that circRNAs and mRNAs in antler mesenchymal and cartilage tissues are correlated with processes such as cartilage development, bone formation, and cell proliferation and differentiation. Differentially expressed circRNAs may participate in the biological processes regulated by mRNAs and play an auxiliary regulatory role. The differential expression of circRNAs and mRNAs between the two tissues may be one of the key factors promoting antler regeneration and proliferation.

Secondly, the binding sites and relationships between circRNAs, miRNAs, and mRNAs were predicted using TargetScan (5.0a) and Miranda (3.3a) bioinformatics analysis software, and the ceRNA network of circRNA-miRNA-mRNA was mapped. The results of the GO analysis revealed that the biological processes of these mRNAs were mainly enriched in chondrocyte differentiation and bone resorption, and the KEGG analysis showed that the mRNAs in the ceRNA network were mainly enriched in osteoclast differentiation, the FOXO signaling pathway, the Toll-like receptor signaling pathway, and other signaling pathways. These results suggest that some circRNAs in this ceRNA network bind to miRNAs through molecular sponge action, in order to regulate the expression of functional genes related to chondrocyte and osteoblast differentiation.

Finally, based on the results of the GO and KEGG enrichment analysis, a ceRNA network related to antler regeneration and development was constructed, and some functional genes were identified, such as MGP, PTH1R, PTH, FOXO4, and ACP5. These genes have been suggested to be related to chondrogenesis, osteogenesis, and angiogenesis in previous studies. Among them, basal MGP is a vitamin K-dependent protein whose main function is to regulate the calcification and ossification of blood vessels and cartilage tissue by binding to Ca^2+^ [21,22]. PTHrP receptor 1 (PTH1R) is a receptor for PTH-related peptide (PTHrP). Parathyroid hormone (PTH) is a hormone that stimulates bone resorption and regulates chondrocyte differentiation during cartilage internalization [23,24]. Wu et al. [25] found that FOXO4 maintained the activity of hUC-MSCs by inhibiting the apoptosis of human umbilical cord mesenchymal stem cells and delaying the senescence of the stem cell population. Acid phosphatase 5 (ACP5) is an evolutionarily conserved multifunctional protein involved in normal bone development and osteoblast regulation, among other functions [26]. PTH can be involved in regulating MGP expression through the MAPK pathway [27]; ACP can interact with p53 to regulate SMAD3 [28] and ACP5 can activate the IGF-1/Akt pathway to regulate cell cycles [29]; and FOXO4 has been reported to be activated by PI3K-AKT to promote cell proliferation [30] and can also protect the viability of senescent cells and inhibit apoptosis by isolating p53 in the nucleosome to protect the viability of senescent cells and inhibit apoptosis [31]. Collectively, these functional genes in the network interact with each other and together participate in the PI3K/ATK pathway, MAPKt pathway, and p53 pathway, which may be key signaling pathways in antler regeneration.

In addition, a circRNA2829 with the most significant differential expression ploidy was screened from the ceRNA network, and it is speculated that circRNA2829 may be one of the important ncRNA molecules. The results indicate that circRNA2829 promoted the proliferation of chondrocyte cells and increased the level of ALP in chondrocytes, which is an early marker of osteoblast differentiation and is expressed in osteogenic precursor cells, and the level of ALP activity can reflect the trend of cell transformation to osteoblasts [32]. RT-qPCR and the Western blot assay again demonstrated that circRNA2829 increased the expression level of osteogenic marker genes (ALP, OCN, BMP2, RUNX2, SOX9). This result pinpoints that circRNA2829 has an important regulatory role in the mesenchymal-to-chondrogenic transformation process. To verify whether circRNA2829 and miR-4286- R+1 have molecular sponge adsorption, mimics and inhibitors of miR-4286-R+1 were transfected in chondrocytes, respectively. The results revealed that the effect of miR-4286-R+1 on chondrocyte cell phenotype was diametrically opposed to the function of circRNA2829. This suggests that circRNA2829 may affect cell proliferation and differentiation by regulating miR-4286-R+1.

Based on this, the direct target of miR-4286-R+1 was predicted to be FOXO4 on the sequencing results, and the interaction relationship and target binding between circRNA2829, miR-4286-R+1 and FOXO4 were further clarified by rescue assays and dual luciferase reporter assays. Ago2 is an indicator protein for circRNAs to exert spongy effects [33]. circRNA2829 and miR-4286-R+1 were found to be effectively down-drawn by anti-Ago2 antibodies by Ago2 RIP assay, but not pulled down by non-specific anti-IgG antibodies. This suggests that circRNA2829 may regulate FOXO4 gene expression through sponge adsorption of miR-4286-R+1, which in turn promotes antler chondrocyte differentiation. It has been shown that the FOXO family maintains skeletal homeostasis and promotes osteogenic differentiation by regulating oxidative stress in osteoblasts, and it has also been reported that the FOXO family has a bidirectional regulation of the osteogenic process in cells [34,35]. On the other hand, the FOXO family can inhibit the differentiation of mesenchymal stem cells to adipocytes by inhibiting PPARγ [36]. Therefore, it is inferred that the low expression of FOXO4 in MSCs may help maintain the dynamic balance of antler cell differentiation, regulating the tendency of MSCs to cartilage differentiation and reducing the differentiation of MSCs to adipocytes. On the contrary, the high expression of FOXO4 in chondrocytes helps to promote osteogenic differentiation and accelerate the rapid growth and differentiation process of antlers, thus completing the regeneration of antlers in an orderly manner.

In conclusion, the whole-transcriptome sequencing and analysis of antler tissues as well as systematic cell phenotypic and functional experiments were used to construct a deer antler tissue ceRNA network and demonstrate that cirRNA2829 regulates the expression of the target gene FOXO4 by interacting with miR-4286-R+1, which in turn promotes the proliferation and differentiation of antler cells. These findings provide new insights into the underlying mechanisms of the regenerative development and rapid growth of the antler.

## 4. Materials and Methods

### 4.1. Sample Collection and Cell Culture

Sika deer antler samples were collected from three three-year-old sika male deer with the same living conditions and good health (for experimental purposes) from the Experimental Deer Farm of Jilin Agricultural University (Changchun, China). Following the method reported by Li [37], the tip mesenchymal and cartilaginous tissues of the antler were separated after cutting off the antler at the top 4–5 cm in the direction perpendicular to the growth axis. Next, the obtained tissues were cut up as much as possible and Collagenase I, Hyaluronidase, and Collagenase II were added sequentially. The samples were centrifuged at 1200 rpm for 5 min and the cells were resuspended using DMEM medium containing 15% FBS (Gibco, Grand Island, NY, USA) and cultured in an incubator at 37 °C with 5% CO_2_.

### 4.2. RNA-Seq and Sequencing Data Analysis

The whole-transcriptome high-throughput sequencing of the antler tip tissues and the analysis of sequencing data were performed by LC Sciences (Hangzhou, China). The QC report and specific steps for library construction and RNA annotation analysis can be found in Supplementary File S4.

### 4.3. Real-Time Quantitative PCR

Total RNA was extracted and RT-qPCR was performed with the SPARKeasy Total RNA Extraction Kit, SPARKscript Ⅱ RT Master Mix, and 2× SYBR Green qPCR Mix (Sparkjade, Shandong, China). β-actin genes were used for mRNA, circRNA, and lncRNA standardization, while the U6 gene was used for miRNA standardization. Relative RNA expression levels were calculated using the 2^−ΔΔCt^ method. All primers were designed using Primer 5.0 and the primer sequence is shown in Supplementary Table S1.

### 4.4. Construction of the ceRNA Networks

The combination relationships and sites of differential expression circRNAs, miRNAs, and mRNAs were predicted and analyzed using TargetScan (5.0a) and Miranda (3.3a) software (TargetScan_score ≥ 90, Miranda_Energy < −20). Next, ceRNA relationship pairs were collected based on common miRNA combinations, and ceRNA network maps were constructed using Cytoscape (3.8.0) software to visualize regulatory and control relationships.

### 4.5. GO and KEGG Pathway Analysis

The gene ontology (GO) enrichment and KEGG pathway analysis for host genes of differential expression circRNA, mRNA, and ceRNA networks were conducted using the KOBAS (3.0) software. The GO terms and KEGG pathway with corrected *p* < 0.05 were significantly enriched.

### 4.6. CircRNA Circular Structure Validation

To confirm the circular structure, splice sequence sequencing, back-to-back primer PCR, RNase R treatment, and actinomycin D treatment were performed. Divergent and convergent circRNA2829 primers were designed to amplify the circular circRNA2829 isolated from chondrocytes. Sanger sequencing was used to verify the back-splicing region. The RNase R treatment experiments were performed by treating chondrocyte RNA with 5 U/mg of RNase R at 37 °C for 30 min. The chondrocytes were treated with actinomycin D (2 mg/mL) and total RNA was extracted from chondrocytes according to the reagent protocol. Thereafter, the stability of the circRNA2829 and linear GAPDH mRNA were investigated by RT-qPCR.

### 4.7. Vector Construction and Cell Transfection

The circRNA2829 overexpression vector (OE-circRNA2829) and small interfering RNA (si-circRNA2829 and si-FOXO4) were designed and synthesized by Hanbio Biotechnology (Shanghai, China), and the miR-4286-R+1 mimic and inhibitor were purchased from RiboBio. The cell transfection was performed using the Lipo8000™ Transfection Reagent (Beyotime, Shanghai, China), according to the manufacturer’s instructions. The transfection was performed up to 48 h, unless otherwise specified, followed by subsequent cell treatments. Experiments were performed three times independently.

### 4.8. Cell Proliferation Assay

Cell proliferation was detected with EdU and CCK-8 assays. The EdU assay was performed according to the protocol of the BeyoClick™ EdU-488. Briefly, the treated cells were seeded in 12-well plates and incubated with 50 μM EdU for 4 h at 37 °C. After being fixed with 4% paraformaldehyde, the cells were exposed to 250 μL of click additive solution and then incubated with 5 μg/mL Hoechst 33342 to stain cell nuclei. Images were captured using a fluorescence microscope (AX10 Carl Zeiss, Jena, Germany). The percentage of EdU-positive cells was defined as the proliferation rate. For the CCK-8 assay, the treated cells were seeded in 96-well plates and incubated with 100 μL 10% CCK-8 solution for 4 h at 37 °C. The absorbance was measured at 450 nm.

### 4.9. Alkaline Phosphatase (ALP) Staining and Alkaline Phosphatase Assay

The staining of cells in each treatment group was detected with the BCIP/NBT kit: cells were seeded in 12-well plates. Transfection was performed when the cell density reached 80%, incubated with a BCIP/NBT color development solution for 4 h, and then the reaction was ended by washing twice with PBS. The ALP activity was detected in cells from different treatment groups using the ALP assay kit: cells were treated with lysate to obtain the supernatant, mixed with assay buffer, incubated for 5–10 min at 37 °C, and OD values were recorded at 450 nm. Finally, the ALP activity of each group of cells was calculated according to the formula in the instructions of the ALP assay kit.

### 4.10. Western Blot Assay

The cell specimens from various groups were lysed by RIPA lysis buffer (Beyotime, Shanghai, China), and the corresponding protein contents were determined by the enhanced BCA protein analysis kit (Bioss, Beijing, China), according to the manufacturer’s guide. Subsequently, equal quantities of the protein of each sample were separated by using 10% or 12% SDS-PAGE and were transferred onto the polyvinylidene fluoride membrane. The membrane was then blocked at 25 °C for 1 h with 5% non-fat dried milk in Tris-buffered saline with Tween (TBST) and incubated with the specific primary antibody (1:500 or 1:1000) overnight at °C. Then the samples were incubated with the horseradish peroxidase (HRP)-conjugated secondary antibody (bs-0295G-HRP/bs-0296G-HRP, Bioss, Beijing, China) at 25 °C for 2 h. The protein expression was observed with enhanced chemiluminescence reagents. The primary antibodies used in the present study are listed as follows: FOXO4 (WL03596, Wanleibio, Shenyang, China), SOX9 (WL02290, Wanleibio, Shenyang, China), RUNX2 (PTM-5253, PTM Bio, Hangzhou, China), BMP2 (WL03816, Wanleibio, Shenyang, China), Osteocalcin (WLH4378, Wanleibio, Shenyang, China), and β-actin (TA811000, ORIGENE, Wuxi, China).

### 4.11. Dual-Luciferase Reporter Assay

The wild type (circRNA2829-wild/FOXO4-wild) and mutant type (circRNA2829-mut/FOXO4-mut) without the miR-4286-R+1 binding site were cloned into the pmiR-RB-Report vector and it was confirmed that the cloning was successful. These plasmids were transfected into miR-4286-R+1 overexpressing chondrocytes, and the dual luciferase activity was measured by lysing cells 48 h after transfection, according to the protocol.

### 4.12. RIP Assay

The RIP assay was performed using an RIP kit (BersinBio, Guangzhou, China) according to the manufacturer’s instructions. Briefly, 4 × 10^7^ cells were lysed in polysome lysis buffer containing protease and RNase inhibitors. They were incubated with RIP buffer containing magnetic beads conjugated with anti-Ago2 antibodies and immunoglobulin G (IgG) antibodies for 16 h at 4 °C. Then, the proteinase K was used to digest the unbound proteins, and the co-immunoprecipitated RNA was isolated. Subsequently, the expression of circRNA2829 and miR-4286-R+1 in each group was detected by RT-qPCR. 

### 4.13. Statistical Analysis

The experimental data were presented as means ± standard deviation (SD). SPSS 22.0 software (IBM, Armonk, NY, USA) was used to perform all the statistical analyses. The significant differences between the groups were evaluated by Student’s *t*-test. *p* values < 0.05 was considered statistically significant.

## Data Availability

All data used in the article can be found in Supplementary Files S1–S3.

## Supplementary Materials

The following supporting information can be downloaded at: https://www.mdpi.com/article/10.3390/ijms24087204/s1. [38,39,40,41,42,43,44,45,46,47,48,49].

## References

[1] Price J., Allen S. (2004). Exploring the mechanisms regulating regeneration of deer antlers. Philos. Trans. R. Soc. London. Ser. B Biol. Sci..

[2] Kierdorf U., Kierdorf H., Szuwart T. (2007). Deer antler regeneration: Cells, concepts, and controversies. J. Morphol..

[3] Barrell G.K., Davies R., Bailey C.I. (1999). Immunocytochemical localization of oestrogen receptors in perichondrium of antlers in red deer (*Cervus elaphus*). Reprod. Fertil. Dev..

[4] Allen S.P., Maden M., Price J.S. (2002). A role for retinoic acid in regulating the regeneration of deer antlers. Dev. Biol..

[5] Sun Z., Ma Y., Chen F., Wang S., Chen B., Shi J. (2018). miR-133b and miR-199b knockdown attenuate TGF-β1-induced epithelial to mesenchymal transition and renal fibrosis by targeting SIRT1 in diabetic nephropathy. Eur. J. Pharmacol..

[6] Wan P., Su W., Zhang Y., Li Z., Deng C., Li J., Jiang N., Huang S., Long E., Zhuo Y. (2020). LncRNA H19 initiates microglial pyroptosis and neuronal death in retinal ischemia/reperfusion injury. Cell Death Differ..

[7] Zheng S., Zhang X., Odame E., Xu X., Chen Y., Ye J., Zhou H., Dai D., Kyei B., Zhan S. (2021). CircRNA-Protein Interactions in Muscle Development and Diseases. Int. J. Mol. Sci..

[8] Xiong J., Zhang H., Wang Y., Cheng Y., Luo J., Chen T., Xi Q., Sun J., Zhang Y. (2022). Rno_circ_0001004 Acts as a miR-709 Molecular Sponge to Regulate the Growth Hormone Synthesis and Cell Proliferation. Int. J. Mol. Sci..

[9] Hansen T.B., Jensen T.I., Clausen B.H., Bramsen J.B., Finsen B., Damgaard C.K., Kjems J. (2013). Natural RNA circles function as efficient microRNA sponges. Nature.

[10] Kristensen L.S., Andersen M.S., Stagsted L.V.W., Ebbesen K.K., Hansen T.B., Kjems J. (2019). The biogenesis, biology and characterization of circular RNAs. Nat. Rev. Genet..

[11] Kulcheski F.R., Christoff A.P., Margis R. (2016). Circular RNAs are miRNA sponges and can be used as a new class of biomarker. J. Biotechnol..

[12] Panda A.C. (2018). Circular RNAs Act as miRNA Sponges. Adv. Exp. Med. Biol..

[13] Sen R., Ghosal S., Das S., Balti S., Chakrabarti J. (2014). Competing endogenous RNA: The key to posttranscriptional regulation. Sci. World J..

[14] Feleke M., Bennett S., Chen J., Hu X., Williams D., Xu J. (2021). New physiological insights into the phenomena of deer antler: A unique model for skeletal tissue regeneration. J. Orthop. Transl..

[15] Chen D.Y., Li Y.J., Jiang R.F., Li Y.T., Feng J., Hu W. (2021). Effects and mechanism of lncRNA-27785.1 that regulates TGF-β1 of Sika deer on antler cell proliferation. J. Cell. Physiol..

[16] Chen D.Y., Yang M., Sun Z.T., Song M.M., Yao H.B., Long G.H., Hu W. (2022). Notch4 affects the proliferation and differentiation of deer antler chondrocytes through the Smad3/lncRNA27785.1 axis. Cell. Signal..

[17] Yan Y., Chen D., Han X., Liu M., Hu W. (2020). MiRNA-19a and miRNA-19b regulate proliferation of antler cells by targeting TGFBR2. Mammal Res..

[18] Jiang Q., Liu C., Li C.P., Xu S.S., Yao M.D., Ge H.M., Sun Y.N., Li X.M., Zhang S.J., Shan K. (2020). Circular RNA-ZNF532 regulates diabetes-induced retinal pericyte degeneration and vascular dysfunction. J. Clin. Investig..

[19] Wang Y., Liu J., Ma J., Sun T., Zhou Q., Wang W., Wang G., Wu P., Wang H., Jiang L. (2019). Exosomal circRNAs: Biogenesis, effect and application in human diseases. Mol. Cancer.

[20] Xue C., Li G., Lu J., Li L. (2021). Crosstalk between circRNAs and the PI3K/AKT signaling pathway in cancer progression. Signal. Transduct. Target. Ther..

[21] Bjørklund G., Svanberg E., Dadar M., Card D.J., Chirumbolo S., Harrington D.J., Aaseth J. (2020). The Role of Matrix Gla Protein (MGP) in Vascular Calcification. Curr. Med. Chem..

[22] Li X., Wei R., Wang M., Ma L., Zhang Z., Chen L., Guo Q., Guo S., Zhu S., Zhang S. (2020). MGP Promotes Colon Cancer Proliferation by Activating the NF-κB Pathway through Upregulation of the Calcium Signaling Pathway. Mol. Ther. Oncolytics.

[23] Cui C., Bi R., Liu W., Guan S., Li P., Song D., Xu R., Zheng L., Yuan Q., Zhou X. (2020). Role of PTH1R Signaling in Prx1(+) Mesenchymal Progenitors during Eruption. J. Dent. Res..

[24] Amano K., Densmore M., Fan Y., Lanske B. (2016). Ihh and PTH1R signaling in limb mesenchyme is required for proper segmentation and subsequent formation and growth of digit bones. Bone.

[25] Wu P.P., Hu W.L., Yin C.C., Fei J.W. (2020). FOXO4 maintains senescence in human umbilical cord mesenchymal stem cells by repressing apoptosis. Sheng Li Xue Bao [Acta Physiol. Sin.].

[26] Ren X., Shan W.H., Wei L.L., Gong C.C., Pei D.S. (2018). ACP5: Its Structure, Distribution, Regulation and Novel Functions. Anti-Cancer Agents Med. Chem..

[27] Suttamanatwong S., Franceschi R.T., Carlson A.E., Gopalakrishnan R. (2007). Regulation of matrix Gla protein by parathyroid hormone in MC3T3-E1 osteoblast-like cells involves protein kinase A and extracellular signal-regulated kinase pathways. J. Cell. Biochem..

[28] Hu Y., Yu J., Wang Q., Zhang L., Chen X., Cao Y., Zhao J., Xu Y., Jiang D., Wang Y. (2020). Tartrate-Resistant Acid Phosphatase 5/ACP5 Interacts with p53 to Control the Expression of SMAD3 in Lung Adenocarcinoma. Mol. Ther. Oncolytics.

[29] Lång P., Patlaka C., Andersson G. (2021). Tartrate-resistant acid phosphatase type 5/ACP5 promotes cell cycle entry of 3T3-L1 preadipocytes by increasing IGF-1/Akt signaling. FEBS Lett..

[30] Gong C., Ai J., Fan Y., Gao J., Liu W., Feng Q., Liao W., Wu L. (2019). NCAPG Promotes The Proliferation of Hepatocellular Carcinoma through PI3K/AKT Signaling. Onco. Targets Ther..

[31] Baar M.P., Brandt R.M.C., Putavet D.A., Klein J.D.D., Derks K.W.J., Bourgeois B.R.M., Stryeck S., Rijksen Y., van Willigenburg H., Feijtel D.A. (2017). Targeted Apoptosis of Senescent Cells Restores Tissue Homeostasis in Response to Chemotoxicity and Aging. Cell.

[32] Sharma U., Pal D., Prasad R. (2014). Alkaline phosphatase: An overview. Indian J. Clin. Biochem..

[33] Schirle N.T., Sheu-Gruttadauria J., MacRae I.J. (2014). Structural basis for microRNA targeting. Science.

[34] Almeida M. (2011). Unraveling the role of FoxOs in bone–insights from mouse models. Bone.

[35] Ma X., Su P., Yin C., Lin X., Wang X., Gao Y., Patil S., War A.R., Qadir A., Tian Y. (2020). The Roles of FoxO Transcription Factors in Regulation of Bone Cells Function. Int. J. Mol. Sci..

[36] Chen D., Gong Y., Xu L., Zhou M., Li J., Song J. (2019). Bidirectional regulation of osteogenic differentiation by the FOXO subfamily of Forkhead transcription factors in mammalian MSCs. Cell Prolif..

[37] Li C., Clark D.E., Lord E.A., Stanton J.A., Suttie J.M. (2002). Sampling technique to discriminate the different tissue layers of growing antler tips for gene discovery. Anat. Rec..

[38] Kechin A., Boyarskikh U., Kel A., Filipenko M. (2017). cutPrimers: A New Tool for Accurate Cutting of Primers from Reads of Targeted Next Generation Sequencing. J. Comput. Biol..

[39] Langmead B., Salzberg S.L. (2012). Fast gapped-read alignment with Bowtie 2. Nat. Methods.

[40] Kim D., Pertea G., Trapnell C., Pimentel H., Kelley R., Salzberg S.L. (2013). TopHat2: Accurate alignment of transcriptomes in the presence of insertions, deletions and gene fusions. Genome Biol..

[41] Pertea M., Pertea G.M., Antonescu C.M., Chang T.-C., Mendell J.T., Salzberg S.L. (2015). StringTie enables improved reconstruction of a transcriptome from RNA-seq reads. Nat. Biotechnol..

[42] Frazee A.C., Pertea G., Jaffe A.E., Langmead B., Salzberg S.L., Leek J.T. (2015). Ballgown bridges the gap between transcriptome assembly and expression analysis. Nat. Biotechnol..

[43] Kong L., Zhang Y., Ye Z.-Q., Liu X.-Q., Zhao S.-Q., Wei L., Gao G. (2007). CPC: Assess the protein-coding potential of transcripts using sequence features and support vector machine. Nucleic Acids Res..

[44] Sun L., Luo H., Bu D., Zhao G., Yu K., Zhang C., Liu Y., Chen R., Zhao Y. (2013). Utilizing sequence intrinsic composition to classify protein-coding and long non-coding transcripts. Nucleic Acids Res..

[45] Trapnell C., Williams B.A., Pertea G., Mortazavi A., Kwan G., Van Baren M.J., Salzberg S.L., Wold B.J., Pachter L. (2010). Transcript assembly and quantification by RNA-Seq reveals unannotated transcripts and isoform switching during cell differentiation. Nat. Biotechnol..

[46] Kim D., Salzberg S.L. (2011). TopHat-Fusion: An algorithm for discovery of novel fusion transcripts. Genome Biol..

[47] Zhang X.-O., Dong R., Zhang Y., Zhang J.-L., Luo Z., Zhang J., Chen L.-L., Yang L. (2016). Diverse alternative back-splicing and alternative splicing landscape of circular RNAs. Genome Res..

[48] Zhang X.O., Wang H.B., Zhang Y., Lu X., Chen L.L., Yang L. (2014). Complementary Sequence-Mediated Exon Circularization. Cell.

[49] Robinson M.D., McCarthy D.J., Smyth G.K. (2010). EdgeR: A Bioconductor package for differential expression analysis of digital gene expression data. Bioinformatics.

