# Supplementary material for: Whole-Transcriptome Sequencing of Antler Tissue Reveals That circRNA2829 Regulates Chondrocyte Proliferation and Differentiation via the miR-4286-R+1/FOXO4 Axis
Source: Int J Mol Sci. 2023 Apr 13;24(8):7204. doi: 10.3390/ijms24087204 (PMC10139046; doi:10.3390/ijms24087204)
Supplement: Supplementary file 1 [file ijms-24-07204-s001.zip › Supplementary table1.pdf]

Table S1. Specific primer sequences for qPCR and RT-PCR.

Gene	Forward primer (5’-3’)	Reverse primer (5’-3’)
circRNA1803	GCACAGTCATCGTCCCCTTT	CAACCTTAACCACGCCATACAC
circRNA3045	CGGCCTGGGATTTAAAGG	GGATGGATGGACAGATGGATAG
circRNA686	CAAACCGAAACACAAGGGAA	GAGTGGGCGTAGGAGGCA
circRNA856	GGCGCAGATGGAACTCAAA	CGAATCAGGCTGAAGTCCATAG
circRNA4658	GCAAGAAGGCGGACACCA	CTTATCAATGACCGCCCG
MSTRG.17242	GCATCTTCTCCACACCCTCAA	TCCACTAGCTTCGCACTCTCAT
MSTRG.19659	GAAGTGAAGCAGCAGGCGA	AGAGCGAGCAAAGGAAGACC
MSTRG.2983	TCAATCTCCATCCTCACCCC	GCTTAAACTCCTCATGCACCTG
MSTRG.27000	AATTGCAGTGCATTTGGGC	TCTGTTCTCGTGAGTTGTGTCG
MSTRG.3522	ATCAACGCCTACCTGCACATC	AGCAAACGCAATCAAGACTCC
mRNA1803	ACCTTTAATTCCGCCACCTT	GCTTTTCACCTCACTTCCCAT
mRNA3045	GAGAGTGCTTGTGTTTCGGTTT	TGAGAAGTGAGGAAAATCGGG
mRNA686	ACAGAACCGATGTGGAGTATGAG	GAGAGTGCTTGTGTTTCGGTTT
mRNA856	CATGACGTTGCACCTTCCAA	TCCTCGTTTCTCTGCTCGC
mNA4658	GACTGCCAACGAATCTGCC	TGTGCCTCTGTATTTCCCGA
β-actin	GCACCCAGCACGATGAAAAT	GACAATGGAGGGTCCGGATT
circRNA2829 for q-PCR	CGAAATGAGGGACCTGTTACC	GACACATCCATCAGGCGTAAA
circRNA2829 convergent	TATGGTCCCTATTCTGATGTCTCA	CTGTAATCGACAGCAATACTGTGC
circRNA2829 divergent	TTACGCCTGATGGATGTGTC	AAGAGATACAAGGTGCTTTGCA
FOXO4	GTGCTGGCGGAAGAGGAAA	CGAGTGGGGAGATGTGAGATTG
OCN	GCAGCGAGGTGGTGAAGAGA	GAAGCCGATGTGGTCAGCTA
BMP2	GTAGGTGGGAGAGCTTCGAT	GGCGTTTCCGCTGTTTATGT
RUNX2	AGCTATTAAAGTCACAGTGGATGG	GGCGATCAGAGAACAAACTAGG
SOX9	CCCCCCACTACCCCCAAAA	CGCTACTCAGTTCGCCGATG
