# Supplementary material for: Whole-Transcriptome Sequencing of Antler Tissue Reveals That circRNA2829 Regulates Chondrocyte Proliferation and Differentiation via the miR-4286-R+1/FOXO4 Axis
Source: Int J Mol Sci. 2023 Apr 13;24(8):7204. doi: 10.3390/ijms24087204 (PMC10139046; doi:10.3390/ijms24087204)
Supplement: Supplementary file 1 [file ijms-24-07204-s001.zip › SupplementaryFile4.pdf]

The sequencing data filter statistics

Sample	Raw reads number	Clean reads number	Clean rate(%)	Q20(%)	Q30(%)	GC content%
C1	95311996	90337590	94.78	98.49	95.72	51
C2	90918926	84334874	92.76	98.47	95.66	54
C3	76365224	70348496	92.12	98.39	95.48	55
M1	67090872	62451958	93.09	98.55	95.89	53
M2	74001608	67794916	91.61	98.45	95.64	53
M3	64430430	59821794	92.85	98.43	95.61	51

RNA extraction and library construction

The total RNA of the two kinds of tissues was extracted by the Trizol method (Invitrogen, CA, USA). Use Nano Drop2000 (Thermo Science) to detect the number of RNA. Finally, Agilent2100 is used to check the integrity of RNA, and the number of RIN is more than 7.0, construction of three libraries for high-throughput sequencing.

RNase R treat library: To get circRNA sequences, after depletion of ribosomal RNA by Ribo-Zero™ rRNA Removal Kit (Illumina, San Diego, USA). RNase R was used to remove linear RNAs and to enrich circRNAs. After removing ribosomal and linear RNAs, the enriched circRNAs were fragmented into small pieces using divalent cations under high temperatures. The cleaved RNA fragments were reverse-transcribed to create the cDNA. The average insert size for the final cDNA library was 300 bp (±50 bp). At last, we performed the paired-end sequencing on an Illumina HiSeqTM 2500 platform (LC Sciences, Hangzhou, China) following the vendor's recommended protocol.

rRNA-depleted RNA library: To get mRNA sequences, approximately 5 μg of total RNA was used to deplete ribosomal RNA according to the manuscript of the Ribo-

Zero™ rRNA Removal Kit (Illumina, San Diego, USA). The RNA fragmentation kit (Ambion) was used to fragment rRNA-depleted RNA. Then the cleaved RNA fragments were reverse-transcribed to create the cDNA. Finally, we performed the paired-end sequencing on an Illumina HiSeqTM 2500 platform (LC Sciences, Hangzhou, China) following the vendor's recommended protocol.

MiRNA library: To establishment miRNA library, Illumina Truseq Small RNA Preparation Kit (Illumina, San Diego, USA) was used for 5μg total RNAs, then sequenced using Illumina HiSeqTM 2500 platform (LC Sciences, Hangzhou, China). HISAT2 2.1.0 was used to map the sample data to the reference genome, The reference genome was obtained from: https://www.ncbi.nlm.nih.gov/genome/?term=Cervus.

RNA annotation and analysis

First of all, the sequencing results of the above three libraries were screened. The raw data obtained by sequencing was screened by Cutadapt [1] software, and the reads pairs with high N content and the reads pairs with low mass and high base content were removed. After filtering out the inferior reads data, the clean reads for subsequent analysis are obtained, and the three libraries are annotated and analyzed respectively.

mRNA: We used Bowtie2 [2] and topaht2 [3] to map reads to the genome of *Cervus elaphus*. The mapped reads of each sample were assembled using StringTie [4]. Then, all transcriptomes from Samples were merged to reconstruct a comprehensive transcriptome using Perl scripts. After the transcriptome was generated, StringTie and Ballgown [5] were used to estimate the expression levels of all transcripts. Transcripts that overlapped with known mRNAs and transcripts shorter than 200 bp were discarded.

Then we utilized CPC [6] and CNCI [7] to predict transcripts with coding potential. All transcripts with CPC score <-1 and CNCI score <0 were removed. The remaining transcripts were considered as lncRNAs. StringTie was used to perform expression levels for mRNA by calculating FPKM [8]. The differentially expressed mRNA were selected with log2 (fold change) >1 or log2 (fold change) <-1 and with statistical significance (p-value < 0.05) by R package – Ballgown.

CircRNA: Cutadapt was used to remove the reads that contained adaptor contamination, low-quality bases, and undetermined bases. The sequence quality was verified using FastQC (http://www.bioinformatics. Babraham. ac. uk/projects /fastqc/). We used Bowtie2 and Tophat2 to map reads to the genome of *Cervus elaphus*. The remaining reads (unmapped reads) were still mapped to the genome using tophat-fusion [9]. CIRCExplorer [10,11] was used to Lenovo assemble the mapped reads to circular RNAs at first; Then, back-splicing reads were identified in unmapped reads by tophat-fusion and CIRCExplorer. All samples generated unique circular RNAs. The differentially expressed circRNAs were selected with log2 (fold change) >1 or log2 (fold change) <-1 and with statistical significance (p-value < 0.05) by R package–edgeR [12].

miRNA: Raw reads were subjected to an in-house program, ACGT101-miR (LC Sciences, Houston, Texas, USA), to remove adapter dimers, junk, low complexity, common RNA families (rRNA, tRNA, snRNA, snoRNA), and repeats. Subsequently, unique sequences with length in 18~26 nucleotide were mapped to *Cervus elaphus* precursors in miRBase 22.0 by BLAST search to identify known miRNAs and novel

3p- and 5p- derived miRNAs. Differential expression of miRNAs based on normalized deep-sequencing counts was analyzed by selectively using Fisher exact test, Chi-squared 2X2 test, Chi-squared nXn test, Student t-test, or ANOVA based on the experiment's design. The significance threshold was set to be 0.01 and 0.05 in each test. To predict the genes targeted by the most abundant miRNAs, two computational target prediction algorithms (TargetScan 50 and Miranda 3.3a) were used to identify miRNA binding sites.

References

1. Kechin A, Boyarskikh U, Kel A, Filipenko M. cutPrimers: A New Tool for Accurate Cutting of Primers from Reads of Targeted Next Generation Sequencing. Journal of computational biology: a journal of computational molecular cell biology. 2017;24(11):1138-43.

2. Langmead B, Salzberg SL. Fast gapped-read alignment with Bowtie 2. Nature methods. 2012;9(4):357-9.

3. Kim D, Pertea G, Trapnell C, Pimentel H, Kelley R, Salzberg SL. TopHat2: accurate alignment of transcriptomes in the presence of insertions, deletions and gene fusions. Genome biology. 2013;14(4):R36.

4. Pertea M, Pertea GM, Antonescu CM, Chang TC, Mendell JT, Salzberg SL. StringTie enables improved reconstruction of a transcriptome from RNA-seq reads. Nature biotechnology. 2015;33(3):290-5.

5. Frazee AC, Pertea G, Jaffe AE, Langmead B, Salzberg SL, Leek JT. Ballgown bridges the gap between transcriptome assembly and expression analysis. Nature

biotechnology. 2015;33(3):243-6.

6. Kong L, Zhang Y, Ye ZQ, Liu XQ, Zhao SQ, Wei L, et al. CPC: assess the protein-coding potential of transcripts using sequence features and support vector machine. Nucleic acids research. 2007;35(Web Server issue):W345-9.

7. Sun L, Luo H, Bu D, Zhao G, Yu K, Zhang C, et al. Utilizing sequence intrinsic composition to classify protein-coding and long non-coding transcripts. Nucleic acids research. 2013;41(17):e166.

8. Trapnell C, Williams BA, Pertea G, Mortazavi A, Kwan G, van Baren MJ, et al. Transcript assembly and quantification by RNA-Seq reveals unannotated transcripts and isoform switching during cell differentiation. Nature biotechnology. 2010;28(5):511-5.

9. Kim D, Salzberg SL. TopHat-Fusion: an algorithm for discovery of novel fusion transcripts. Genome biology. 2011;12(8):R72.

10. Zhang XO, Dong R, Zhang Y, Zhang JL, Luo Z, Zhang J, et al. Diverse alternative back-splicing and alternative splicing landscape of circular RNAs. Genome research. 2016;26(9):1277-87.

11. Zhang XO, Wang HB, Zhang Y, Lu X, Chen LL, Yang L. Complementary sequence-mediated exon circularization. Cell. 2014;159(1):134-47.

12. Robinson MD, McCarthy DJ, Smyth GK. edgeR: a Bioconductor package for differential expression analysis of digital gene expression data. Bioinformatics (Oxford, England). 2010;26(1):139-40.
